# Haploinsufficiency of myostatin protects against aging-related declines in muscle function and enhances the longevity of mice

**DOI:** 10.1111/acel.12339

**Published:** 2015-03-24

**Authors:** Christopher L Mendias, Konstantin I Bakhurin, Jonathan P Gumucio, Mark V Shallal-Ayzin, Carol S Davis, John A Faulkner

**Affiliations:** 1 Department of Orthopaedic Surgery, University of Michigan Medical SchoolAnn Arbor, MI, USA; 2 Department of Molecular & Integrative Physiology, University of Michigan Medical SchoolAnn Arbor, MI, USA

**Keywords:** GDF-8, longevity, muscle atrophy, muscle contractility, myostatin, sarcopenia, skeletal muscle

## Abstract

The molecular mechanisms behind aging-related declines in muscle function are not well understood, but the growth factor myostatin (*MSTN*) appears to play an important role in this process. Additionally, epidemiological studies have identified a positive correlation between skeletal muscle mass and longevity. Given the role of myostatin in regulating muscle size, and the correlation between muscle mass and longevity, we tested the hypotheses that the deficiency of myostatin would protect oldest-old mice (28–30 months old) from an aging-related loss in muscle size and contractility, and would extend the maximum lifespan of mice. We found that *MSTN*^+/−^ and *MSTN*^−/−^ mice were protected from aging-related declines in muscle mass and contractility. While no differences were detected between *MSTN*^+/+^ and *MSTN*^−/−^ mice, *MSTN*^+/−^ mice had an approximately 15% increase in maximal lifespan. These results suggest that targeting myostatin may protect against aging-related changes in skeletal muscle and contribute to enhanced longevity.

Sarcopenia is the pathological loss in muscle mass and strength that occurs with aging (Gumucio & Mendias, 2013). In mice, muscle mass and force production slowly decreases from adulthood (6–9 months of age) to old age (22–24 months), with a rapid deterioration present once mice reach oldest-old ages (>26–28 months) (Brooks & Faulkner, 1988; Lynch *et al*., 2001; Graber *et al*., 2013). There is also an aging-associated increase in collagen accumulation which can diminish force production (Ramaswamy *et al*., 2011). In humans, muscle mass is positively correlated with a greater longevity (Miller *et al*., 2002), and the rapid decrease in muscle mass and strength that occurs toward the end of the lifespan can lead to severe disability and reduced quality of life (Fielding *et al*., 2011).

Myostatin is a negative regulator of skeletal muscle mass, with adult *MSTN*^−/−^ mice displaying up to a twofold increase in muscle mass (Gumucio & Mendias, 2013). Myostatin induces atrophy by upregulating the E3 ubiquitin ligases atrogin-1 and MuRF-1 and by inhibiting the IGF-1 pathway (Gumucio & Mendias, 2013). As the role of myostatin in regulating muscle function in oldest-old mice had not previously been studied, and there is a positive correlation between muscle mass and longevity in humans (Miller *et al*., 2002), we tested the hypotheses that oldest-old male myostatin-deficient mice would have improved muscle force production compared to wild-type mice and that the deficiency of myostatin would increase the maximum lifespan of mice.

Circulating myostatin protein was not detectable in *MSTN*^−/−^mice, while *MSTN*^+/−^ mice had a 30% decrease (Table S1). For the fast-fibered EDL, *MSTN*^+/−^ and *MSTN*^−/−^ mice had a greater mass (Fig.1A) and number of type II muscle fibers (Fig. S1) than controls. Maximum isometric force production (P_o_) was increased in *MSTN*^+/−^ and *MSTN*^−/−^ mice (Fig.1B), although no differences in specific force production (sP_o_), which is P_o_ normalized to muscle cross-sectional area (CSA), were noted (Fig.1C). Atrogin-1 was decreased in *MSTN*^−/−^ mice, but no other differences in MuRF-1 were observed (Fig.1D–E).

**Fig 1:**
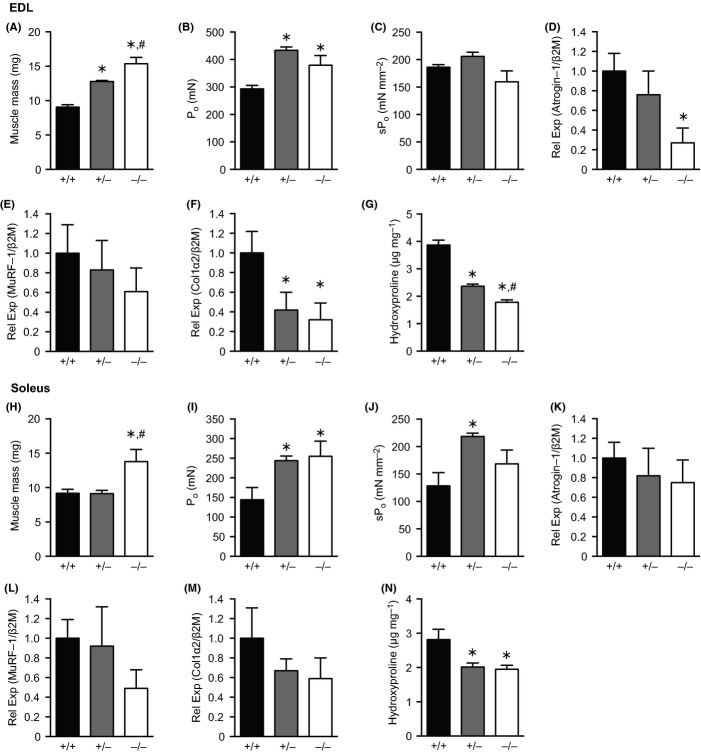
Muscle contractility, hydroxyproline and gene expression values of EDL muscles (A through G) and soleus muscles (H through N) from 28- to 30-month old MSTN^+/+^, MSTN^+/−^ and MSTN^−/−^ mice. (A, H): Wet mass. (B, I): Maximum isometric force (P_o_). C, J: Specific force (sP_o_). Gene expression for (D, K) Atrogin-1, (E, L) MuRF-1 and (F, M) Type 1 collagen. G, N: Hydroxyproline content. Values are mean ± SE; *N* = 6 mice per genotype. Differences tested with a one-way ANOVA and Fisher’s LSD post hoc sorting. *, different from *MSTN*^+/+^ (*P* < 0.05). ^#^, different from *MSTN*^+/−^ (*P* < 0.05).

For mixed-fiber soleus muscles, *MSTN*^−/−^ mice had increased mass (Fig.1H). No change in the percent distribution of fiber types or fiber CSA was observed, although there was an increase in the number of fibers in *MSTN*^+/−^ and *MSTN*^−/−^ mice (Fig. S1). Interestingly, despite both *MSTN*^+/−^ and *MSTN*^−/−^ mice demonstrating a substantial increase in P_o_ (Fig.1I), only the *MSTN*^+/−^ mice had an increase in sP_o_ (Fig.1J). No differences in atrogin-1 or MuRF-1 expression were observed (Fig.1K–L). The differences between muscle mass and P_o_ across the three genotypes are also similar to previous reports in adult animals, but sP_o_ was only elevated in adult *MSTN*^−/−^ mice (Mendias *et al*., 2006) unlike in the current study. Combined, these results suggest the prolonged deficiency of myostatin protects against the aging-associated decrease in P_o_ without having a negative impact on sP_o_ in oldest-old mice. Further, as fiber loss is considered to be the primary contributor to aging-associated muscle atrophy (Gumucio & Mendias, 2013), there appears to be a protective effect of myostatin deficiency on the primary cause of aging-related muscle weakness.

We next evaluated changes in the muscle ECM, as myostatin can directly induce collagen expression in muscle and fibroblast cells (Mendias *et al*., 2006, 2008). Hydroxyproline, which is a marker of collagen, and type I collagen expression were reduced in EDL muscles of *MSTN*^+/−^ and *MSTN*^−/−^ mice (Fig.1F–G). For soleus muscles, *MSTN*^+/−^ and *MSTN*^−/−^ mice had a reduction in hydroxyproline, although no change in type I collagen expression was detected (Fig.1M–N). This reduction in collagen is consistent with findings in adult animals (Mendias *et al*., 2006), and the reduction in collagen levels in oldest-old mice may contribute to their improved contractile properties.

In the lifespan study, there were no differences between the survival curves of *MSTN*^+/+^ and *MSTN*^−/−^ mice, but *MSTN*^+/−^ mice had significant different survival curves from *MSTN*^+/+^ and *MSTN*^−/−^ mice (Fig.2A). *MSTN*^+/−^ mice also had an increase in maximal lifespan and maximum age (Fig.2B). Approximately 2/3 of mice could be submitted to necropsy, and the only pathological finding of significance was gross cardiomegaly. No differences in relative heart mass were present between *MSTN*^+/+^ and *MSTN*^+/−^ mice, but there was an increase observed in *MSTN*^−/−^ mice (Fig.2B). Although we did not evaluate female mice in this study, we do not anticipate sex-specific differences in these findings.

**Fig 2:**
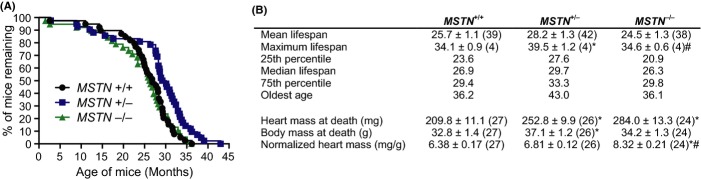
Lifespan data for MSTN^+/+^, MSTN^+/−^ and MSTN^−/−^ mice. (A) Survival curves of mice. Log-rank analysis of survival curves: *MSTN*^+/+^ vs. *MSTN*^−/−^, *P* = 0.85; *MSTN*^+/+^ vs. *MSTN*^+/−^, *P* = 0.003; *MSTN*^+/−^ vs. *MSTN*^−/−^, *P* = 0.003. (B) Summary longevity and cardiac data. Values are mean ± SE (N). Differences tested with a one-way ANOVA and Fisher’s LSD post hoc sorting. *, different from *MSTN*^+/+^ (*P* < 0.05). ^#^, different from *MSTN*^+/−^ (*P* < 0.05).

Mouse strains with loss of function in the growth hormone/IGF-1 axis have a smaller body size and enhanced lifespan (Blagosklonny, 2013). The current results are the first to identify a loss of function gene mutation in mice that results in an increase in muscle and body mass along with enhanced longevity. The mechanism behind the increased longevity of *MSTN*^+/−^ mice is not known, but inhibition of myostatin can reduce systemic inflammatory proteins and body fat (Gumucio & Mendias, 2013). Given the increase in relative heart mass, the contribution of aging-associated cardiomegaly to mortality (Lakatta & Levy, 2003) and that inhibition of myostatin can increase heart mass (Bish *et al*., 2011), it is possible that positive effects of increased skeletal muscle mass on the longevity of *MSTN*^−/−^ mice was offset by cardiac pathologies.

Most genetic models of enhanced longevity in mice have identified an inverse relationship between body mass and longevity, which has lead to the observation that ‘big mice die young’ (Blagosklonny, 2013). However, the results from the current study support the epidemiological observations in humans that when it comes to skeletal muscle mass and longevity, bigger may be better.

## Supporting Information

**Data S1** Experimental procedures.

**Fig. S1** Histology for EDL muscles (A through C) and soleus muscles (D through H) from 28 to 30 month old *MSTN*^+/+^, *MSTN*^+/−^ and *MSTN*^−/−^ mice.

**Table S1** Morphological and contractile properties measurements.

**Table S2** Lifespan data.
