# Supplementary material for: Haploinsufficiency of myostatin protects against aging-related declines in muscle function and enhances the longevity of mice
Source: Aging Cell. 2015 Mar 24;14(4):704–6. doi: 10.1111/acel.12339 (PMC4531085; doi:10.1111/acel.12339)
Supplement: Supplementary file 1 [file acel0014-0704-sd1.pdf]

Data S1 - Experimental Procedures

Animals. This study was approved by the University of Michigan IACUC. Animal handling and care was performed in accordance with US Public Health Service Policy on Humane Care and Use of Laboratory Animals. Myostatin-deficient mice (*MSTN*$^{-/-}$), in which the myostatin gene was inactivated by replacing a portion of the third exon of the *MSTN* gene which encodes the mature myostatin peptide with a *Neo* cassette (McPherron *et al.* 1997), were kindly provided by Dr. Se-Jin Lee. The background strain of the mice was C57BL/6. Animals were housed in ventilated micro-isolator cages under specific pathogen free conditions, and water and a standard Lab Diet 5001 chow (Purina Lab Diet, St. Louis, MO) ad libidum. Equal 12 hour light/dark cycles were maintained in the facility. Mice were housed up to four per cage with 1/8 Bed-O-Cobs bedding (Andersons Lab Bedding, Maumee, OH). Pups were weaned three weeks after birth, at which time an ear tag was placed and a small tail biopsy for obtained to analyze *MSTN* genotype by PCR as previously described (Mendias *et al.* 2006).

Lifespan Study. A lifespan study of 39 wild type (*MSTN*$^{+/+}$), 42 heterozygous null (*MSTN*$^{+/-}$) and 38 homozygous null (*MSTN*$^{-/-}$) male mice was conducted using a previously described study design (Chamberlain *et al.* 2007). The American Foundation for Aging Research guidelines for use of animals in gerontological research were followed (Miller & Nadon 2000). Mice were monitored daily after weaning and dates were recorded for all mice that died spontaneously throughout the study. In rare cases, mice that showed signs of distress such as visible tumors, listlessness or that stopped eating and were deemed close to death, were humanely euthanized. All mice in the lifespan study that were found dead or euthanized, and did not display signs of excessive autolysis or cannibalism were placed in a container of 10% neutral

buffered formalin and transferred to the University of Michigan Unit for Laboratory Animal Medicine for necropsy.

Surgical Procedure. Male mice, 28-30 months of age, were used for the measurement of contractile properties, histology and biochemistry. Mice were placed in a deep anesthesia using isoflurane. The right extensor digitorum longus (EDL) and soleus muscles were used for contractility measurements and histology, while the left muscles were used for hydroxyproline content and gene expression analysis. For muscles used in contractility experiments, a 5-0 silk suture was tied to the proximal and distal tendons of EDL and soleus muscles, just past the aponeuroses. Muscles were then removed from the animal and placed immediately in bath that contained Krebs mammalian Ringer solution supplemented with 0.25 mM tubocurarine chloride maintained at 25°C, and bubbled with a mixture of 95% O_2 and 5% CO_2 to maintain a pH of 7.4. Muscles that were removed for biochemistry were removed, weighed, finely minced with scissors, divided into two equal portions, and prepared for hydroxyproline assays or RNA isolation. Following removal of muscles, a cardiac puncture was performed to remove blood, and mice were humanely euthanized by removal of the heart.

Measurement of Contractile Properties. Contractile properties of muscles was performed as previously described (Mendias *et al.* 2006). The distal tendon of the muscle was attached to a servomotor (Aurora Scientific, Aurora, ON) and the proximal tendon attached to a force transducer (Kulite Semiconductor, Leonia, NJ). Muscles were stimulated by square wave pulses delivered from platinum electrodes connected to a high power current stimulator (Aurora Scientific). A PC and custom designed software (LabVIEW, National Instruments, Austin, TX) recorded data from the force transducer at 20 kHz and also controlled pulse properties and servomotor activity. The voltage of pulses was increased, and the muscle length was adjusted to

the length (L_o) that resulted in maximum twitch force (P_t). L_o was measured with digital calipers. Muscles were held at L_o and stimulated with trains of pulses to generate isometric contractions. Pulse trains were 300 ms for EDL muscles and 900 ms for soleus muscles. Stimulus frequency was increased until the maximum isometric force (P_o) was achieved. To calculate specific maximum isometric force (sP_o), P_o was divided by the physiological cross section area (PCSA), which was determined by dividing the muscle mass by the product of L_f and $1.06 g/cm^3$, the density of mammalian skeletal muscle (Brooks & Faulkner 1988).

Following measurement of maximum twitch force and isometric contractility, a mechanical injury was produced by subjecting muscles to two 40% lengthening contractions. Muscles were stimulated and held at L_o for 100 ms for EDL muscles and 300 ms for soleus muscles to allow for the development of P_o. Following the isometric contraction, muscles were stretched through a 40% strain relative to L_f at a velocity of 1 L_f/s. After the active stretch, muscles were returned to L_o, remained inactive for 1 min, then were subjected to a second 40% active stretch. At the completion of contractile properties measurements, the muscle was taken out of the bath, the sutures were removed, and the muscle was weighed and prepared for histology.

Histology. Muscles were blotted dry and snap frozen in TissueTek in isopentane cooled with liquid nitrogen. Muscles were cryosectioned into 10μm sections and blocked with a Mouse on Mouse Blocking Kit (Vector Labs, Burlingame, CA) and incubated with primary antibodies against type I myosin heavy chain (A4.840 IgM, Developmental Studies Hybridoma Bank, Iowa City, IA), type II myosin heavy chain (My32 IgG, Thermo Scientific, Rockford, IL) and collagen I (biotinylated Ab65777, AbCam, Cambridge, MA), as well as highly cross-adsorbed fluorescent tagged secondary antibodies AF555 goat anti-mouse IgM (Invitrogen, Grand Island, MY),

AF350 goat-anti mouse IgG (Invitrogen) and fluorescein conjugated streptavidin (Vector Labs). Sections were mounted in Prolong Gold (Invitrogen) and imaged using a Axiophot microscope (Zeiss, Thonwood, NY) equipped with an AxioCam camera (Zeiss). CyteSeer software (Vala Sciences, San Diego, CA) and ImageJ (NIH, Bethesda, MD) were used for quantitative histomorphometry. Four random fields under the 20× objective were analyzed per muscle in a blinded fashion.

Hydroxyproline Assay. Hydroxyproline content of muscles was measured as previously described (Woessner 1961; Mendias *et al.* 2006). Muscles were dried for two hours at 110ºC and then hydrolyzed in 500µL of 6 M hydrochloric acid overnight at 130ºC. The muscle hydrolysate was neutralized with an equal volume of 6 M sodium hydroxide. A standard curve was constructed using known amounts of purified L-hydroxyproline (Sigma, St. Louis, MO). Samples were assayed in triplicate using a SpectraMax microplate reader (Molecular Devices, Sunnyvale, CA). Values are presented in µg of hydroxyproline normalized to the dry mass of the muscle in mg.

Gene Expression. RNA was isolated from muscle using an RNeasy Fibrous Tissue Kit (Qiagen, Valencia, CA) and treated with DNase I (Qiagen). RNA was reverse transcribed into cDNA using an Omniscript RT system (Qiagen) and oligo-dT$_{15}$ primers. cDNA was amplified in a CFX96 real time thermal cycler (BioRad, Hercules, CA) using a SYBR Green I PCR system (Qiagen) along with custom primers listed below. Cycle threshold (C_t) values of type Iα2 collagen (Col1α2), atrogin-1 and muscle ring finger-1 (MuRF-1) were normalized to the stable housekeeping gene β2-microglobulin (β2M) using the $2^{-\Delta\Delta CT}$ method (Schmittgen & Livak 2008). The presence of single amplicons from qPCR reactions was verified using melting curve analysis. Primer sequences for Col1α2, atrogin-1 and B2M have previously been reported

(Gumucio *et al.* 2013), and sequences for MuRF-1 were 5'- GTGTGAGGTGCCTACTTGCTC-3' (forward) and 5'-GCTCAGTCTTCTGTCCTTGGA-3' (reverse).

Measurements of Serum Myostatin. Blood was collected from mice via cardiac puncture, and allowed to coagulate for 30 minutes at room temperature prior to centrifugation at approximately 1000×g for 10 minutes to separate serum. Myostatin levels were measured in duplicated using an ELISA kit (R&D Systems, Minneapolis, MN) following manufacturer instructions, and absorbance values were read in a SpectraMax microplate reader.

Statistical Analysis. Data are presented as mean±SE. A one-way ANOVA was used to determine the differences between three *MSTN* genotypes. Fisher's LSD post hoc sorting was used to identify differences between specific groups ($P<0.050$). Student's t-test ($P<0.050$) was used to determine differences in serum myostatin levels between $MSTN^{+/+}$ and $MSTN^{+/-}$ mice, as myostatin was not detectable in $MSTN^{-/-}$ mice. Log-rank tests were used to evaluate differences between survival curves, and to account for multiple comparisons significance was adjusted from 0.050 to 0.016 using the Bonferroni method. All analysis was performed in Prism 6.0 (GraphPad Software, La Jolla, CA).

References

Brooks SV, Faulkner JA (1988). Contractile properties of skeletal muscles from young, adult and aged mice. *J Physiol (Lond)*. **404**, 71-82.

Chamberlain JS, Metzger J, Reyes M, Townsend D, Faulkner JA (2007). Dystrophin-deficient mdx mice display a reduced life span and are susceptible to spontaneous rhabdomyosarcoma. *FASEB J*. **21**, 2195-2204.

Gumucio JP, Flood MD, Phan AC, Brooks SV, Mendias CL (2013). Targeted inhibition of TGF-beta results in an initial improvement but long-term deficit in force production after contraction-induced skeletal muscle injury. *J Appl Physiol (1985)*. **115**, 539-545.

McPherron AC, Lawler AM, Lee SJ (1997). Regulation of skeletal muscle mass in mice by a new TGF-beta superfamily member. *Nature*. **387**, 83-90.

Mendias CL, Marcin JE, Calerdon DR, Faulkner JA (2006). Contractile properties of EDL and soleus muscles of myostatin-deficient mice. *J Appl Physiol*. **101**, 898-905.

Miller RA, Nadon NL (2000). Principles of animal use for gerontological research. *J Gerontol A Biol Sci Med Sci*. **55**, B117-123.

Schmittgen TD, Livak KJ (2008). Analyzing real-time PCR data by the comparative C(T) method. *Nat Protoc*. **3**, 1101-1108.

Woessner JF (1961). The determination of hydroxyproline in tissue and protein samples containing small proportions of this imino acid. *Arch Biochem Biophys*. **93**, 440-447.
