# Supplementary material for: Haploinsufficiency of myostatin protects against aging-related declines in muscle function and enhances the longevity of mice
Source: Aging Cell. 2015 Mar 24;14(4):704–6. doi: 10.1111/acel.12339 (PMC4531085; doi:10.1111/acel.12339)
Supplement: Supplementary file 2 [file acel0014-0704-sd2.pdf]

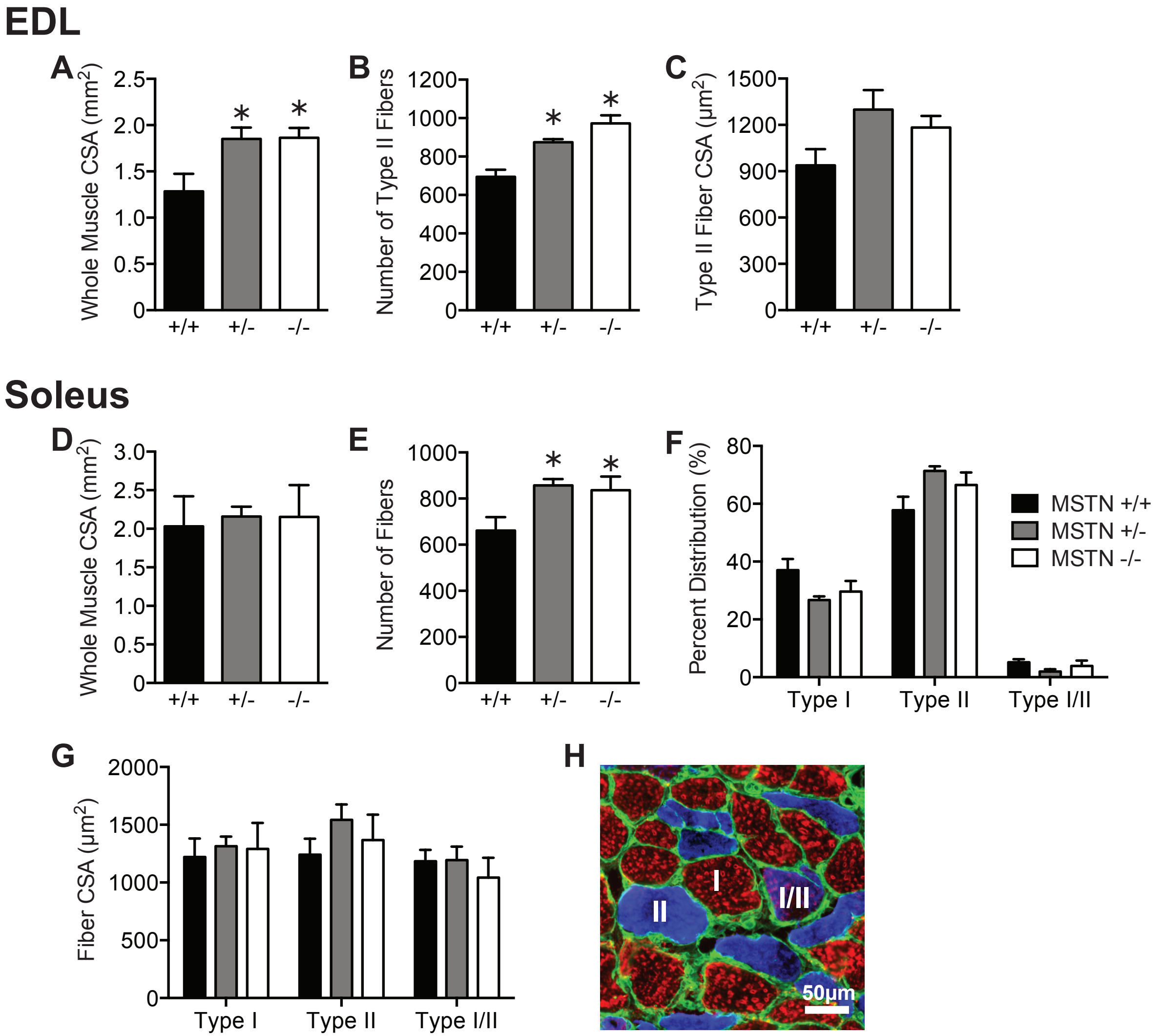

Supplemental Figure 1. Histology for EDL muscles (A through C) and soleus muscles (D through H) from 28-30 month old $\mathit{MSTN}^{+/+}$, $\mathit{MSTN}^{+/-}$ and $\mathit{MSTN}^{-/-}$ mice. For EDL, the whole muscle cross-sectional area (CSA, A), number of type II muscle fibers (B) and the size of type II muscle fibers (C) is shown. For soleus, the whole muscle CSA (D), total number of muscle fibers (E), percent distribution of different fiber types (F) and the size of different fiber types is shown. A representative immunofluorescent section is shown (H), with type I muscle fibers in red, type II muscle fibers in blue, hybrid type I/II fibers in pink and type I collagen in green. Values are mean±SE; N=6 mice per genotype. Differences between groups were tested with a one-way ANOVA and Fisher's LSD post-hoc sorting. *, significantly different from $\mathit{MSTN}^{+/+}$ (P<0.05). #, significantly different from $\mathit{MSTN}^{+/-}$ (P<0.05).
