# Supplementary material for: Haploinsufficiency of myostatin protects against aging-related declines in muscle function and enhances the longevity of mice
Source: Aging Cell. 2015 Mar 24;14(4):704–6. doi: 10.1111/acel.12339 (PMC4531085; doi:10.1111/acel.12339)
Supplement: Supplementary file 3 [file acel0014-0704-sd3.pdf]

| | $MSTN^{+/+}$ | $MSTN^{+/-}$ | $MSTN^{-/-}$ |
|---|---|---|---|
| Body mass (g) | 33.5±0.3 | 37.2±0.9 | 36.6±1.8 |
| Serum myostatin (ng/mL) | 8.00±0.45 | 5.65±0.23* | ND |
| *EDL Muscles* | | | |
| Wet mass (mg) | 9.1±0.3 | 12.8±0.2* | 15.4±0.9*# |
| $L_f$ (mm) | 5.4±0.1 | 5.7±0.1 | 5.9±0.1* |
| $L_o$ (mm) | 12.4±0.3 | 13.0±0.2 | 13.5±0.2* |
| PCSA ($mm^2$) | 1.6±0.1 | 2.1±0.1* | 2.5±0.1*# |
| $P_t$ (mN) | 64.3±12.6 | 130.8±8.2* | 127.0±9.9* |
| $sP_t$ ($mN/mm^2$) | 40.0±7.1 | 62.1±4.2 | 53.3±6.0 |
| dP/dt (mN/ms) | 5.7±1.2 | 11.4±0.8* | 12.2±1.1* |
| TTPT (ms) | 22.4±1.9 | 24.3±2.6 | 18.6±2.2 |
| 1/2 RT (ms) | 27.3±2.0 | 16.1±0.5* | 15.5±0.6* |
| $P_o$ (mN) | 293.1±12.8 | 433.6±12.2* | 379.5±34.7* |
| $sP_o$ ($mN/mm^2$) | 186.2±4.8 | 205.9±7.5 | 159.7±19.8 |
| Stretch 1 Force Deficit (% of $P_o$) | 13.6±1.4 | 14.3±0.9 | 14.1±1.3 |
| Stretch 2 Force Deficit (% of $P_o$) | 30.9±5.0 | 29.2±1.6 | 28.4±2.1 |
| *Soleus Muscles* | | | |
| Wet mass (mg) | 9.2±0.6 | 9.1±0.5 | 13.8±1.8*# |
| $L_f$ (mm) | 7.9±0.1 | 7.7±0.2 | 8.2±0.1 |
| $L_o$ (mm) | 11.1±0.1 | 10.9±0.3 | 11.5±0.2 |
| PCSA ($mm^2$) | 1.1±0.1 | 1.1±0.1 | 1.6±0.2*# |
| $P_t$ (mN) | 23.6±5.2 | 42.7±0.8 | 45.6±10.0 |
| $sP_t$ ($mN/mm^2$) | 21.5±4.4 | 38.5±1.8 | 30.9±6.7 |
| dP/dt (mN/ms) | 1.2±0.2 | 2.8±0.1* | 3.1±0.7* |
| TTPT (ms) | 41.1±5.9 | 29.2±0.5* | 27.6±1.8* |
| 1/2 RT (ms) | 56.2±5.9 | 35.0±1.4* | 33.9±3.9* |
| $P_o$ (mN) | 144.3±31.3 | 244.0±11.7* | 255.0±38.5* |
| $sP_o$ ($mN/mm^2$) | 128.4±24.2 | 218.4±6.1* | 168.3±25.1 |
| Stretch 1 Force Deficit (% of $P_o$) | 20.4±2.4 | 15.5±1.9 | 18.9±4.4 |
| Stretch 2 Force Deficit (% of $P_o$) | 23.0±4.1 | 19.0±2.4 | 24.0±5.8 |

***Supplemental Table 1. Morphological and contractile properties measurements.*** Values are mean±SE; N=6 mice per genotype. For all measures except serum myostatin levels, differences were tested using a one-way ANOVA followed by Fisher LSD post-hoc sorting. For serum myostatin levels, since myostatin was not detectable (ND) in *MSTN*$^{-/-}$ mice, we performed a t-test. *, significantly different from *MSTN*$^{+/+}$ (P<0.05). #, significantly different from *MSTN*$^{+/-}$ (P<0.05).
