# Supplementary material for: Haploinsufficiency of myostatin protects against aging-related declines in muscle function and enhances the longevity of mice
Source: Aging Cell. 2015 Mar 24;14(4):704–6. doi: 10.1111/acel.12339 (PMC4531085; doi:10.1111/acel.12339)
Supplement: Supplementary file 4 [file acel0014-0704-sd4.pdf]

**MSTN$^{+/+}$**		***MSTN$^{+/-}$***		***MSTN$^{-/-}$***	
Mouse	Age at Death (mo)	Mouse	Age at Death (mo)	Mouse	Age at Death (mo)
1	2.6	1	2.8	1	1.5
2	11.0	2	8.9	2	2.6
3	14.3	3	9.1	3	8.9
4	14.6	4	12.0	4	12.4
5	19.5	5	12.9	5	15.6
6	20.5	6	15.6	6	16.3
7	21.6	7	17.6	7	17.3
8	22.6	8	23.9	8	18.2
9	22.9	9	26.4	9	19.9
10	23.6	10	27.6	10	21.2
11	23.8	11	27.6	11	22.1
12	24.4	12	27.7	12	23.3
13	24.7	13	28.0	13	23.4
14	24.9	14	28.3	14	24.4
15	25.0	15	28.3	15	25.5
16	25.4	16	28.3	16	25.6
17	25.6	17	28.5	17	25.7
18	26.3	18	28.6	18	25.8
19	26.8	19	28.9	19	26.1
20	26.9	20	28.9	20	26.4
21	27.7	21	29.2	21	26.8
22	28.1	22	30.1	22	27.1
23	28.2	23	30.3	23	27.1
24	28.4	24	30.9	24	27.7
25	28.4	25	31.0	25	27.9
26	28.8	26	31.4	26	28.2
27	29.2	27	31.6	27	28.5
28	29.2	28	32.0	28	29.4
29	29.3	29	32.4	29	29.6
30	29.4	30	32.9	30	30.5
31	29.5	31	32.9	31	30.8
32	30.5	32	33.3	32	30.8
33	30.7	33	33.4	33	31.7
34	31.6	34	33.8	34	32.9
35	31.7	35	34.1	35	33.4
36	32.0	36	34.7	36	33.8
37	33.5	37	35.9	37	35.1
38	34.6	38	36.2	38	36.1
39	36.2	39	37.5		
		40	38.4		
		41	39.1		
		42	43.0		

Supplemental Table 2. Lifespan data.
